# Regional Variability in the Prevalence of Food Insecurity and Diet Quality among United States Children

**DOI:** 10.3390/nu16020224

**Published:** 2024-01-10

**Authors:** Jayna M. Dave, Tzuan A. Chen, Alexandra N. Castro, Mamie White, Elizabeth A. Onugha, Sloane Zimmerman, Debbe Thompson

**Affiliations:** 1 USDA/ARS Children’s Nutrition Research Center, Department of Pediatrics, Baylor College of Medicine, 1100 Bates Avenue, Houston, TX 77030, USA; alexandra.castro@bcm.edu (A.N.C.); mawhite@bcm.edu (M.W.); dit@bcm.edu (D.T.); 2 Department of Psychological, Health, and Learning Sciences, University of Houston, 3657 Cullen Boulevard, Houston, TX 77204, USA; tchen3@central.uh.edu; 3 HEALTH Research Institute, University of Houston, 4349 Martin Luther King Boulevard, Houston, TX 77204, USA; 4 Renal Services, Texas Children’s Hospital, 1102 Bates Avenue, Houston, TX 77030, USA; eaonugha@texaschildrens.org; 5 Department of Pediatrics—Nephrology, Baylor College of Medicine, 1102 Bates Avenue, Houston, TX 77030, USA; 6 Department of Pediatrics—Gastroenterology, Baylor College of Medicine, One Baylor Plaza, Houston, TX 77030, USA; sloane.zimmerman@bcm.edu

**Keywords:** food insecurity, diet quality, urban-rural differences, children, federal nutrition assistance program, NHANES

## Abstract

Understanding the association between food security status (FSS) and diet quality in children is crucial. This study investigated regional variability in FSS, participation in the federal nutrition assistance program (FNAP), and diet quality among US children. National Health and Nutrition Examination Survey (NHANES) data from 2013 to 2016 were analyzed. The association between FSS, FNAP participation, and diet quality (Healthy Eating Index—HEI-2015) was assessed using multiple linear/logistic regression models. The sample included 6403 children (mean age: 7.5 years; 51% male; 33% Hispanic). Within the sample, 13% reported child food insecurity, and 30% reported household food insecurity. Additionally, 90% participated in the FNAP, and 88% were enrolled in school lunch programs. Children in urban areas were significantly more likely to report household food insecurity than those in rural areas (29.15% vs. 19.10%). The overall HEI-2015 score was 48.2. The associations between child/household FSS and FNAP participation as well as between child/household FSS and diet quality did not differ by urban/rural residence status, irrespective of the children’s age groups. There is a need for improvement in children’s diet quality, regardless of age or urban/rural residence. The findings suggest that improving children’s diets requires broader action as well as the prioritizing of children in urban areas experiencing food insecurity in future dietary interventions.

## 1. Introduction

Diet plays a vital role in children’s overall health and well-being. A healthy diet provides children with the nutrients they need to grow and develop. Urban–rural differences in diet among US children have been well-documented, typically showing that rural children consume less nutritious diets than urban children [1,2,3,4]. 

Food security, the access by all people at all times to enough food for an active, healthy life, is one of the several conditions necessary for a population to be healthy and well-nourished [5]. Food insecurity, on the other hand, refers to the limited or uncertain availability of nutritionally adequate and safe foods and is strongly related to poverty [6]. As a result, food insecurity is associated with poor nutrient intake and increases the risk of obesity, diabetes, cardiovascular disease, anxiety, and depression [7,8,9,10]. In 2022, 12.8% of US households and 17.3% of households with children were food-insecure [6]. Households with incomes near or below the federal poverty line (i.e., below $24,600 annually for a household of four), single parents, individuals living alone, Black- and Hispanic-headed households, and households in suburban and rural areas experience higher rates of food insecurity than the national average [6]. Food insecurity also tends to be more prevalent in households with older children (5–17 years) than in households with younger children (0–4 years) [11]. 

Studies of children living in rural communities have shown a pattern of food consumption that is high in fat and sugar, and low in fruit and vegetables [12,13]. However, these studies lack a simultaneously obtained nationally representative sample of both urban and rural children. This limitation prevents an assessment of the relationship between urban/rural status and dietary behaviors. In addition, child poverty rates among children are higher in rural communities compared to their urban counterparts (~26% vs. 20%) [14]. Given the positive association between poverty and food insecurity, it is likely that food insecurity rates may be higher among rural children compared to urban children. However, no studies have specifically investigated the urban–rural differences in food security status among children.

The federal nutrition assistance programs (FNAPs) administered by the US Department of Agriculture aim to provide low-income households with access to food, promote a nutritious diet, offer nutrition education, and increase food security [5]. The five largest FNAPs intended to combat hunger and food insecurity are the Supplemental Nutrition Assistance Program (SNAP), the National School Lunch Program (NSLP), the Special Supplemental Nutrition Program for Women, Infants, and Children (WIC), the School Breakfast Program (SBP), and the Summer Food Service Program (SFSP). In 2022, approximately 46.7% of SNAP households, 41.9% of households receiving free or reduced-price school lunches, and 34.6% of WIC households experienced food insecurity [11]. Among households with children, food insecurity is most prevalent in low-income households that participate in SNAP and receive free or reduced-price lunches [11]. This suggests that those households with the greatest need for food assistance are more likely to participate in FNAP. Several studies have shown a significant association between participation in FNAP and lower rates of food insecurity in households with children, even after accounting for selection bias [15,16,17,18,19]. However, a USDA report showed a decrease in participation in all five major FNAPs between October 2016 and November 2017 [5], suggesting the need for the continued assessment of FNAP participation’s impact on food insecurity. Federal data also revealed that rural SNAP participation rates are significantly greater than the urban participation rates [18,20]. 

Children from low-income, food-insecure households report poorer quality diets than those from households with higher-incomes. Food insecurity is linked to lower intakes of nutrient-dense foods such as fruits, vegetables, and whole grains and higher intakes of energy-dense foods [7,8,21]. These dietary patterns may lead to a net positive energy balance, increasing the risk of obesity and other diet-related chronic diseases. Moreover, households living in low-income neighborhoods, both urban and rural, may encounter difficulties in accessing affordable and nutritious foods [22,23]. Many low-income neighborhoods, particularly in rural areas, lack access to food retailers and are identified as food deserts, where fresh and affordable foods are in short supply [23]. This directly affects the dietary intake of children from low-income households who tend to purchase high-energy-dense and low-nutrient-dense foods [7,8,21]. Thus, it is imperative to continue to examine and understand how one’s food security status relates to children’s dietary intake, particularly in the context of a growing body of literature linking food insecurity to an increased risk of obesity.

Although the determinants of food insecurity in the US are well-researched, gaps in the literature exist, particularly concerning the urban–rural divide. Our research sought to fill this gap, examining the urban–rural differences in and associations between food security status, participation in FNAP, and dietary intake among children across different age groups, i.e., 2–5 years, 6–11 years, and 12–17 years of age.

## 2. Materials and Methods

### 2.1. Participants

This study employed data from the National Health and Nutrition Examination Survey (NHANES) from 2013 to 2016. A secondary analysis of the NHANES data was deemed exempt from review by the Baylor College of Medicine’s Institutional Review Board. The NHANES, administered by the National Center for Health Statistics (NCHS) of the Centers for Disease Control and Prevention (CDC), is a cross-sectional, nationally representative survey of the non-institutionalized civilian population of the US. Details about each survey and their sampling designs can be found elsewhere [24]. Data from two survey cycles (2013–2014 and 2015–2016) containing information on food security status and dietary intake were employed to enhance the reliability and stability of estimates across subgroups [24]. The 2013–2016 NHANES included data on a total of 6685 children aged 2–17 years. Children between 2 and 17 years of age with reported food security status were included in the study. The participants with missing information on any of the independent or dependent variables were excluded from the analysis. The final sample included 6403 children, categorized into three age groups: 2–5 years old, 6–11 years old, and 12–17 years old. The NCHS Ethics Review Board of the CDC approved the NHANES protocols; all the participants provided informed consent prior to data collection.

### 2.2. Measures

#### 2.2.1. Sociodemographic Variables

The sociodemographic variables in the NHANES were collected during the in-home interviews, using the computer-assisted personal interview system. Individuals who were at least 16 years old and emancipated minors were interviewed directly, while proxies provided information for survey participants under the age of 16. The demographic variables included age, gender, race/ethnicity, parent’s marital and education status, and household income. The rural/urban status of the county of residence was determined based on its Urban Influence Code (UIC), as defined by the USDA’s Economic Research Service. These codes were drawn from the 2013 Area Resource File, containing information about the characteristics of the 2142 counties in the US. Counties with UIC codes 1 or 2 were coded as urban, while those with other UIC codes (3–12, designated as nonmetropolitan codes by the USDA’s ERS) were classified as rural [25,26]. The county of residence’s urban–rural status is a restricted-use variable, and these data were accessed through the Research Data Center.

#### 2.2.2. Food Security Status

The food security status was measured using the 18-item US Household Food Security Survey Module during the in-home interviews [27]. An adult provided responses to the 10 items pertaining to the entire household. An additional eight items were specific to households with children aged ≤17 years. Each question referenced the previous 12-month period. The data for food security status (household and child level) were coded using the USDA’s coding guide. A score of 0–18 was created by summing the affirmative responses to the 18 questions, with higher scores indicating worse food insecurity. Consistent with the USDA’s definitions and the existing literature, if the primary household respondent provided two or more affirmative responses to the child-specific questions, the children in the household were classified as food insecure [28]. The experiences of the entire household or children in the household were categorized into one of four food security categories (i.e., high, marginal, low, and very low) based on NHANES documentation [29]. High food security and marginal food security were merged as one category of food security; low and very low food security were merged to classify food insecurity.

#### 2.2.3. Diet Quality—Healthy Eating Index-2015

A single 24 h dietary recall was collected for each participant using the USDA’s automated multiple-pass method. For the participants aged <6 years old, a proxy answered the questions; for the children 6–8 years old, a proxy answered the questions with the child’s assistance; the 9–11-year-old children answered the questions with the assistance of a proxy; and the participants aged 12 years or older answered the questions themselves. Diet quality was assessed using the Healthy Eating Index (HEI-2015) [30], which evaluates how closely an individual’s dietary intake adheres to the Dietary Guidelines for Americans 2015–2020 [31,32]. The HEI-2015 was computed using the basic HEI scoring algorithm based on day 1 of the 24 h dietary recall data from the NHANES. The recall data were collected by trained interviewers. Only the weekday dietary data were employed to explore the association with HEI-2015. Furthermore, only the day 1 recall was utilized, due to a higher non-response rate and individuals tending to report a lower consumption on day 2 (possibly due to under-reporting or survey fatigue). This approach of utilizing only the day 1 recall has been used in previously published studies [33,34,35]. Additional information on the 24 h dietary recall procedure can be found elsewhere [36].

The HEI-2015 is a composite measure of thirteen dietary elements with nine adequacy components (i.e., total fruits, whole fruits, total vegetables, greens and beans, whole grains, dairy, total protein foods, seafood and plant proteins, and fatty acids) and four moderation components (i.e., refined grains, sodium, added sugars, and saturated fats). Detailed information about the HEI-2015 can be found elsewhere [30,37]. The scores on each component sum to a maximum score of 100. Higher scores for the adequacy components indicate greater intakes. For the moderation components, higher scores indicate lower intakes, as lower intakes are considered more desirable. For all the components, a higher score indicates a better-quality diet. A higher total score indicates a greater alignment of the diet with the Dietary Guidelines. HEI scores of <51, 51–80, and >80 indicate “poor diet”, “needs improvement”, and “good diet”, respectively [37,38,39].

#### 2.2.4. Federal Nutrition Assistance Program Participation

The FNAP includes SNAP, WIC, NSLP, SBP, SFSP, or any combination of these programs. SNAP, WIC, NSLP, SBP, SFSP participation was assessed with the following questions: “Do you/Does any member of your household currently receive SNAP or Food Stamp benefits?”; “Is (SP) now receiving benefits from the WIC program?”; “(Do you/Does SP) get these lunches free, at a reduced price, or (do you/does he/she) pay full price?”; “(Do you/Does SP) get these breakfasts free, at a reduced price, or (do you/does he/she) pay full price?”; and “(Do you/Does SP) get a free or reduced price meal at any summer program (he/she) attends?”. The households with low socioeconomic statuses (SES) were eligible to participate in SNAP; if there were children attending school in these households, they were also eligible for free or reduced-price school meals. To address this, four FNAP participation categories were established. Participation in FNAP was then classified as either no participation or any participation. 

#### 2.2.5. Covariates

The covariates included age, sex, race/ethnicity, weight status, and family monthly poverty index. The primary respondent’s age, sex, and race/ethnicity (Non-Hispanic White, Non-Hispanic Black, Hispanic, and Other Race) were reported in the NHANES Demographic Variables and Sample Weights Module, while their family’s monthly poverty level index was included in the NHANES Income Questionnaire Module. The family monthly poverty level index, serving as an indicator for SES, represents a ratio of self-reported monthly income to poverty threshold. This index was chosen as it theoretically offers an equivalent SES measure that can be applied across different age groups. It serves as an economic measure to determine eligibility for certain federal benefits and programs (e.g., NSLP). Moreover, children from households with a family monthly poverty level index less than 1.85 are eligible for participation in some FNAP, including reduced-price lunches [40,41]. Body Mass Index (BMI), expressed as weight in kilograms divided by height in meters squared (kg/m^2^), is a widely employed method for categorizing weight status. The cutoff criteria are based on the CDC’s sex-specific 2000 BMI-for-age growth charts for the US [42]. One’s weight status is categorized as underweight (BMI < 5th percentile), normal weight (BMI 5th to <85th percentile), overweight (BMI 85th to <95th percentile), and obese (BMI ≥ 95th percentile). The underweight children’s data were combined with the normal weight group’s data because of the small sample size for underweight children. All the covariates were self-reported and selected as potential confounding factors in the association between food insecurity and diet quality.

### 2.3. Data Analysis

The descriptive statistics of the participants’ characteristics were computed for three age groups (i.e., 2–5, 6–11, and 12–17 years old). Rao-Scott Chi-square tests were conducted to examine the association of the participants’ household/child food security status and urban/rural residence status for the overall sample and by the age groups. The analysis utilized sample-weighted data to accommodate the complex survey sampling methods (e.g., survey non-response, and post-stratification adjustment) used in the NHANES.

The final outcome models utilized the SAS SURVEYREG or SURVEYFREQ procedure to accommodate the complex, stratified, multistage probability cluster sampling design. In these final outcome models, the associations between participants’ food security status and FNAP participation and the diet quality measures (including HEI-2015 and its 13 subcomponents) were assessed using multiple linear/logistic regression models. In addition, whether these associations were affected by the urban/rural residence status were examined by including the interaction term of the urban/rural residence and food security status. If significant interactions were detected, post hoc analyses were conducted. The covariates included in the final outcome model were age, sex, race/ethnicity, and family monthly poverty index. Those covariates and variables of interest were obtained directly or calculated from the NHANES datasets. For each outcome of interest (i.e., HEI-2015 and its 13 subcomponents), separate final outcome models were run, both for the entire sample and across the three age groups. All the analyses were performed using SAS 9.4. Statistical significance was designated at *p* < 0.05.

## 3. Results

Among the 6403 children included in the study, 27.33% were 2–5 years old, while 40.43% were 6–11 years old, and 32.23% were 12–17 years old (Table 1). The sex distribution was 51% males and 49% females, and most of the children were Hispanic (30.97–33.80%) and underweight or normal weight (59.03–71.95%) across each of the age groups. The reported child food insecurity ranged from 9.66% among the 2–5-year-old children to 14.77% in the 12–17-year-olds; the household food insecurity ranged from 26.19% among the 2–5-year-olds to 29.42% in the 12–17-year-olds. About 89% participated in SNAP, and about 90% participated in school meals programs. 

A significant urban–rural difference was found in the household food security status among the children aged 6–11 years old (*p* = 0.003) (Table 2). The children living in urban areas were more likely to experience household food insecurity compared to the children living in rural areas (29.149% vs. 19.104%). However, there was no significant difference in child food security status by urban–rural residence.

The moderation effects of urban/rural residence status on the association between child/household food security status and the receipt of FNAP were not significant overall and across different children age groups.

Overall, the children had a poor diet quality (HEI-2015) of 48.17 ± 13.14 (Table 3). The children who were 2–5 years old had a slightly higher HEI-2015 score of 52.16 ± 12.97 compared to the children who were 6–11 years old (47.54 ± 12.90) and 12–17 years old (46.04 ± 12.92). However, this difference was not statistically significant. There were no significant differences in the HEI-2015 component scores by age groups. Additionally, the moderation effects of urban/rural residence status on the associations between child/household food security status and diet quality (i.e., HEI and 13 subcomponents) were not significant overall and across different children age groups. In other words, regardless of the children’s age groups, the associations between child/family food security status and diet quality (i.e., HEI and 13 subcomponents) did not vary by urban–rural residency. 

## 4. Discussion

To our knowledge, this is one of the first studies to examine urban–rural differences in the association between food security status, participation in FNAP, and diet quality among children. An important finding of the study was the significant difference in household food security status among children aged 6–11 years, with urban-dwelling children displaying a higher prevalence of food insecurity compared to their rural counterparts. This divergence sheds light on the critical role of residence location in influencing household food security, particularly for this age group. However, no significant differences were observed in child food security status based on urban–rural residence, suggesting that the challenges of food insecurity may be more pronounced at the household level in urban areas for children in the 6–11 years age range. This aligns with a recent report highlighting that food insecurity was most prevalent in principal cities located within large urban areas compared to rural areas [43]. Nevertheless, this finding is contrary to some previous research findings [44,45,46], revealing that children living in rural areas have higher rates of food insecurity than children living in urban areas. However, it is also important to note that the difference in food insecurity status between urban and rural children was not statistically significant after controlling for other factors, such as household income and race/ethnicity. This suggests that the urban–rural difference in food insecurity may be due to other factors, such as access to healthy food and transportation. In fact, a recent study found that most families of teenagers drove a distance of 10 miles or less to grocery shop at least once a week [47]. Nonetheless, this disparity warrants the need for targeted interventions to address food security challenges, particularly in urban environments.

One key aspect of the study was to assess whether urban–rural residence status moderates the relationship between child/household food security status and participation in FNAP. However, no significant moderating effect was observed, both overall and when examined across different age groups of children. This suggests that the influence of food security on participation in these programs is consistent regardless of residence status. This study finding also indicates the importance of FNAP in addressing food security challenges and that FNAP may be effectively reaching children in need of food assistance across different geographic regions and age groups. In fact, federal data from 2017 indicate that, among those eligible for SNAP, participation rates are higher in rural areas (90%) relative to urban areas (82%), and this disparity in participation rates continues to widen [48].

One of the concerning findings of this study was the overall poor diet quality, as indicated by the HEI-2015 scores. While there were slight variations in the scores across different age groups, ranging from 46 to 52 out of 100, these differences were not statistically significant. Furthermore, this study found no significant disparities in the HEI-2015 component scores based on the different age groups, emphasizing the uniformity in diet quality challenges experienced by children across various age brackets, regardless of their urban or rural residence. Moreover, the scores tended to decrease with age, indicating poor adherence to the US Dietary Guidelines among children, regardless of age or geographic residence. In fact, the findings suggested that children showed the lowest adherence to recommendations for the consumption of greens and beans, while demonstrating the highest adherence to guidelines for total protein foods and dairy intake. Previous research using nationally representative samples has found similar results for children’s diet quality in the US [49,50]. These study results underscore the significance of implementing strategies to enhance diet quality among US children, focusing on improving the access to and affordability of nutritious foods [51].

This study also explored whether urban–rural residence moderated the relationship between diet quality and food security status among children, a dimension not previously explored in depth. Interestingly, no significant moderation effects were detected, indicating that the impact of food security status on diet quality remains consistent regardless of residence. While previous research has primarily assessed the relationship between food security status on urban–rural residence and dietary intake, few studies have utilized a nationally representative sample to assess the relationship between urban–rural residency and dietary intake among children. These studies have yielded mixed results, with some reporting no significant differences in dietary intake [52] and others noting slight variations in fruit consumption between urban and rural adolescents [53]. Moreover, Euler et al. [2] found that differences in reported dietary intake based on measures of rurality were minimal once SES and ethnicity were considered. This suggests that differences might be influenced by other community-level access factors or other social determinants of health. These findings suggest that strategies aimed at improving diet quality among children experiencing food insecurity should be implemented uniformly, irrespective of geographic location or age group.

This study’s strengths lie in its utilization of a large nationally representative sample of the US population, which allows for robust conclusions regarding the associations being studied. The NHANES utilizes valid and reliable measures of diet, along with a standardized protocol for assessing weight and height. However, this study is constrained by its cross-sectional design, preventing causal inference. Another limitation is the lack of information regarding the timing of households’ experiences of food insecurity relative to when they received food and nutrition assistance program(s) benefits. This limits the use of statistical analyses to tackle the issue of self-selection bias, a common problem in studies involving FNAP participation [54]. While our models accounted for potential confounding variables, there is a possibility that unmeasured or unobserved confounding factors could impact both FNAP and diet quality. This may partially explain the association between FNAP and diet quality. The assessment of dietary intake relied on a single 24 h dietary recall. This method is susceptible to bias and error due to day-to-day variation in eating habits and the tendency for people to underreport their food intake—a common issue in dietary intake data. Moreover, there is a potential for bias in diet assessment methods attributed to the utilization of a single dietary recall and the likelihood of measurement errors associated with self-reported dietary intake. Another limitation is the absence of an examination of differences in sex, race, or weight status in this study; however, they are important covariates and should be examined in future research. Despite the use of 2013–2016 data, diet quality was assessed using HEI-2015 instead of HEI-2010. Nonetheless, this is not a cause of concern, as multiple updates to school meal guidelines in 2012 align with HEI-2015 [55,56]. 

## 5. Conclusions

In conclusion, these findings underscore the complex interplay of factors affecting food security, diet quality, and the influence of geographic location. While differences were noted in household food security among children aged 6–11 years old, other aspects, including child food security, FNAP participation, and diet quality, exhibited uniform patterns across urban and rural settings and various age groups. These results emphasize the need for comprehensive, age-appropriate interventions to address food security and dietary challenges among children, with a focus on promoting healthier eating habits and reducing the risk of nutrition-related health issues across diverse demographic and geographic contexts.

## Figures and Tables

**Table 1:** Descriptive statistics of participant characteristics by age group.

	All *n* = 6403	2–5 Years Old *n* = 1750	6–11 Years Old *n* = 2589	12–17 Years Old *n* = 2064
	Mean ± SD			
Age	7.51 ± 5.25	3.38 ± 1.14	8.44 ± 1.72	14.44 ± 1.68
BMI Percentile	19.81 ± 5.44	16.49 ± 1.85	18.79 ± 4.31	23.84 ± 6.19
Ratio of Family income to Poverty	1.88 ± 0.92	1.84 ± 0.91	1.87 ± 0.92	1.94 ± 0.92
	N (%)			
Gender				
Male	3266 (51.01)	903 (51.60)	1311 (50.64)	1052 (50.97)
Female	3137 (48.99)	847 (48.40)	1278 (49.36)	1012 (49.03)
Weight Status				
Underweight/Normal weight	3893 (63.70)	1172 (71.95)	1547 (62.05)	1174 (59.02)
Overweight	1048 (17.15)	250 (15.35)	431 (17.29)	367 (18.45)
Obese	1170 (19.15)	207 (12.71)	515 (20.66)	448 (22.52)
Ethnicity				
Non-Hispanic White	1730 (27.02)	486 (27.77)	698 (26.96)	546 (26.45)
Non-Hispanic Black	1556 (24.30)	432 (24.69)	632 (24.41)	492 (23.84)
Hispanic	2111 (32.97)	542 (30.97)	875 (33.80)	694 (33.62)
Others	1006 (15.71)	290 (16.57)	384 (14.83)	332 (16.08)
Child Food Security Status				
Child food security	5480 (87.08)	1552 (90.34)	2202 (86.35)	1726 (85.23)
Child food insecurity	813 (12.92)	166 (9.66)	348 (13.65)	299 (14.76)
Household Food Security Status				
Household food security	4535 (72.04)	1268 (73.81)	1837 (72.01)	1430 (70.58)
Household food insecurity	1760 (27.96)	450 (26.19)	714 (27.99)	596 (29.42)
Participation in Federal Nutrition Assistance Programs				
SNAP	2129 (89.68)	673 (90.21)	875 (89.74)	581 (88.97)
WIC	479 (49.33)	479 (49.33)	NA	NA
SBP	2143 (92.57)	187 (94.92)	1253 (92.95)	703 (91.30)
NSLP	2606 (87.66)	218 (92.77)	1407 (88.60)	981 (85.30)
SFSP	640 (34.32)	49 (35.51)	354 (34.40)	237 (33.95)
Any of the FNAP	3722 (72.60)	938 (77.78)	1619 (71.83)	1165 (69.89)

Notes. SNAP: Supplemental Nutrition Assistance Program; WIC: Women, Infants, and Children; SBP: School Breakfast Program; NSLP: National School Lunch Program; SFSP: Summer Food Service Program; BMI: Body Mass Index; SD: Standard deviation; NA: Not applicable.

**Table 2:** Urban–rural differences in food security status by different age groups.

	Urban			Rural			
Age Group	*n*	%	Weighted %	*n*	%	Weighted %	*p*-Value
Household Food Security status							
All							0.06
Household food security	3834	71.13	76.78	640	79.01	81.09	
Household food insecurity	1556	28.87	23.22	170	20.99	18.91	
2–5 years old							0.13
Household food security	1094	72.93	77.40	156	81.67	84.04	
Household food insecurity	406	27.07	22.60	35	18.33	15.96	
6–11 years old							<0.01
Household food security	1541	70.85	76.68	271	80.89	82.57	
Household food insecurity	634	29.15	23.32	64	19.11	17.43	
12–17 years old							0.54
Household food security	1199	69.91	76.47	213	75.00	78.54	
Household food insecurity	516	30.09	23.53	71	25.00	21.46	
Child Food Security Status							
All							0.94
Child food security	4681	86.88	89.37	717	88.52	89.24	
Child food insecurity	707	13.12	10.63	93	11.48	10.76	
2–5 years old							0.44
Child food security	1350	90.00	91.60	176	92.15	93.53	
Child food insecurity	150	10.00	8.40	15	7.85	6.47	
6–11 years old							0.39
Child food security	1869	85.97	88.15	299	89.25	90.04	
Child food insecurity	305	14.03	11.85	36	10.75	9.96	
12–17 years old							0.33
Child food security	1462	85.30	89.12	242	85.21	86.63	
Child food insecurity	252	14.70	10.88	42	14.79	13.37	

**Table 3:** Healthy Eating Index-2015 component and total scores by age group.

	Maximum Points	All *n* = 6403	2–5 Years Old *n* = 1750	6–11 Years Old *n* = 2589	12–17 Years Old *n* = 2064
		Mean ± SD			
Total HEI-2015 score	100	48.17 ± 13.14	52.16 ± 12.97	47.57 ± 12.90	46.04 ± 12.92
Total Vegetables	5	2.19 ± 1.60	2.07 ± 1.56	2.18 ± 1.57	2.28 ± 1.64
Greens and Beans	5	1.02 ± 1.87	1.03 ± 1.87	1.00 ± 1.85	1.03 ± 1.89
Total Fruits	5	2.54 ± 2.10	3.30 ± 1.98	2.48 ± 2.03	2.08 ± 2.11
Whole Fruits	5	2.30 ± 2.29	2.90 ± 2.28	2.28 ± 2.25	1.90 ± 2.25
Whole Grains	10	2.73 ± 3.29	3.02 ± 3.34	2.76 ± 3.23	2.51 ± 3.32
Dairy	10	6.66 ± 3.35	7.50 ± 3.16	6.68 ± 3.23	6.05 ± 3.49
Total Protein Foods	5	3.60 ± 1.57	3.42 ± 1.59	3.61 ± 1.53	3.71 ± 1.59
Seafood and Plant Proteins	5	1.62 ± 2.09	1.55 ± 2.06	1.66 ± 2.09	1.63 ± 2.12
Fatty Acids	10	3.99 ± 3.55	3.61 ± 3.49	3.91 ± 3.44	4.33 ± 3.67
Sodium	10	4.78 ± 3.37	5.53 ± 3.32	4.85 ± 3.29	4.19 ± 3.38
Refined Grains	10	4.97 ± 3.73	5.71 ± 3.67	4.69 ± 3.62	4.75 ± 3.81
Saturated Fats	10	5.41 ± 3.44	5.38 ± 3.40	5.31 ± 3.43	5.53 ± 3.49
Added Sugars	10	6.36 ± 3.27	7.14 ± 3.00	6.16 ± 3.24	6.05 ± 3.38
Energy kcal		1831.96 ± 821.58	1497.47 ± 585.40	1891.89 ± 740.56	1999.38 ± 966.03

Note: SD: Standard deviation.

## Data Availability

The data analysis used publicly available data that can be accessed at https://wwwn.cdc.gov/nchs/nhanes/continuousnhanes/default.aspx?BeginYear=2013 (accessed on 31 January 2019).
